# Research on Material Removal Mechanism of C/SiC Composites in Ultrasound Vibration-Assisted Grinding

**DOI:** 10.3390/ma13081918

**Published:** 2020-04-19

**Authors:** Dongpo Wang, Shouxiang Lu, Dong Xu, Yuanlin Zhang

**Affiliations:** 1 School of Mechanical Engineering and Automation, Beihang University, Beijing 100191, China; wangdongpo2003@163.com; 2 Key Laboratory for Precision and Non-Traditional Machining Technology of Ministry of Education, Dalian University of Technology, Dalian 116024, China; lushouxiang11@126.com; 3 School of Automation Science and Electrical Engineering, Beihang University, Beijing 100191, China; zyl@buaa.edu.cn

**Keywords:** C/SiC composites, ultrasound vibration, material removal mechanism

## Abstract

C/SiC composites are the preferred materials for hot-end structures and other important components of aerospace vehicles. It is important to reveal the material removal mechanism of ultrasound vibration-assisted grinding for realizing low damage and high efficiency processing of C/SiC composites. In this paper, a single abrasive particle ultrasound vibration cutting test was carried out. The failure modes of SiC matrix and carbon fiber under ordinary cutting and ultrasound cutting conditions were observed and analyzed. With the help of ultrasonic energy, compared with ordinary cutting, under the conditions of ultrasonic vibration-assisted grinding, the grinding force is reduced to varying degrees, and the maximum reduction ratio reaches about 60%, which means that ultrasonic vibration is beneficial to reduce the grinding force. With the observation of cutting debris, it is found that the size of debris is not much affected by the $a_{p}$ with ultrasound vibration. Thus, the ultrasound vibration-assisted grinding method is an effective method to achieve low damage and high efficiency processing of C/SiC composites.

## 1. Introduction

Carbon fiber reinforced silicon carbide composite material (C/SiC) is an advanced composite material with thermal structures. It combines superior mechanical properties of carbon fibers with good chemical and thermal stability of a ceramic matrix. It has high specific strength, good wear resistance, good oxidation resistance, good mechanical properties under high temperature, etc. [1,2]. Compared with a silicon carbide ceramic, the fracture toughness of C/SiC composite is significantly improved due to the introduction of fiber in the ceramic matrix as a barrier for crack propagation. Compared with high-temperature alloys, the working temperature of C/SiC composite increases by 300–500 ${}^{\circ }$C, and the structural weight reduces by 50–70%. C/SiC composite is currently the preferred material for hot-end structural components, high temperature and heat resistant components, and wear-resistant components of aerospace vehicles [1,3,4,5,6,7,8,9,10,11,12,13].

Due to the extremely rough surface and the low dimensional accuracy, it is necessary to perform a cutting process on C/SiC composite material to enhance its quality before the assembling operation. However, C/SiC composite has high hardness, poor thermal conductivity, good wear resistance, anisotropy, and some other characteristics. During the cutting process, some problems such as large cutting force, severe surface damage, and low tool life usually occur [1,14], which seriously affected the production cycle and performance of the composite. Therefore, low damage and high efficiency processing of C/SiC composite has become a bottleneck problem which is needed to be solved in engineering applications.

In recent years, ultrasound vibration-assisted grinding methods have been increasingly used in the processing of hard and brittle materials such as ceramic materials and composite materials. Many researchers have carried out theoretical and experimental studies on wear of the abrasive wheel [15,16], trajectory of the abrasive particle [17,18], grinding force [19,20], and formation mechanism of the machining damage [21,22] of ultrasound vibration-assisted grinding. Results show that ultrasound vibration contributes to reducing cutting forces and tool wear [23,24]. Nevertheless, material properties have a significant impact on the effectiveness of ultrasound vibration-assisted processing, especially for composite materials with complex components and structures. Research on material removal mechanism of ultrasound vibration processing for special materials can lay a theoretical foundation for improvement of the processing technology. However, there are few reports on material removal mechanism of ultrasound vibration-assisted grinding for C/SiC composites, and research have focused on ultrasonic vibration assisted milling of C/SiC composites [25,26].

A single abrasive particle cutting test is an effective method to simplify the grinding process and reveal the material removal mechanism. In order to reveal the ultrasound vibration-assisted processing mechanism, researchers have performed ultrasound vibration cutting tests on different materials, and compared the results of ultrasound cutting with ordinary cutting, to reveal the characteristics of ultrasound vibration-assisted processing. Zhang et al. [27] performed an ultrasound vibration-assisted cutting test on sapphire using a spherical indenter. Results show that ultrasound vibration can reduce the cutting force, effectively avoid microcrack growth, and increase the proportion of plastic deformation. Cao et al. [28] performed ultrasound vibration-assisted cutting tests on SiC ceramics using a tool with single diamond. Results show that ultrasound cutting can improve the cutting critical depth of ductile domain and increase the stiffness of the processing system, and cutting efficiency of the tool is also improved. Feng et al. [29] and Zha et al. [30] compared the cutting forces, friction coefficients, and scratch morphologies of SiCp/Al composites with high volume fraction during ultrasound vibration-assisted cutting and ordinary cutting. Results show that the cutting force and friction coefficient are smaller, and the material removal rate is higher under the condition of ultrasound vibration. From the literature review above, we can conclude that, although ultrasound cutting tests for pure materials and composite materials have been conducted, similar tests for C/SiC composite materials have not yet been performed.

In this paper, tests for ultrasound vibration-assisted cutting with single abrasive particle are performed on C/SiC composites with the comparison of ordinary cutting. The typical machined surface morphologies with different fiber orientation angles are analyzed. By comparing the morphologies of abrasive debris under ordinary cutting and ultrasound cutting conditions, the material removal mechanism of ultrasound vibration-assisted grinding is studied.

## 2. Experimental Setup

### 2.1. Test Material

**Figure 1:**
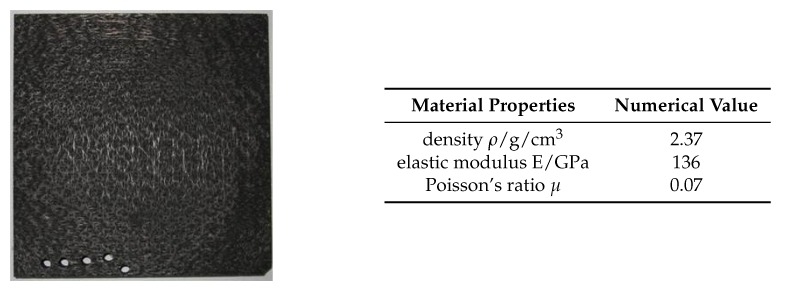
C/SiC test piece and its material properties.

The C/SiC composite material used in the cutting experiments adopts a structure iterating in the order as follows: single-layer $0^{\circ }$ carbon fiber non-woven cloth, SiC matrix layer, $90^{\circ }$ carbon fiber non-woven cloth, and SiC matrix layer. The carbon fiber layer is unidirectional, and short fibers are distributed in the layer due to existence of the mesh inside the SiC matrix layer. To make the whole structure, the relay acupuncture technique is used to vertically pierce the short fibers inside the mesh layer into the non-woven cloth [31]. The needling structure can increase the interlayer strength of C/SiC composites, but the ratio of needling area is relatively small and can be ignored during the cutting process. Figure 1 shows the C/SiC test piece and its material properties. The C/SiC composite was divided into samples with a size of 35 mm× 8 mm× 5 mm, and then the upper surface was polished into a mirror surface to observe the scratches of the single diamond abrasive particle.

### 2.2. Test Devices and Cutting Parameters

**Figure 2:**
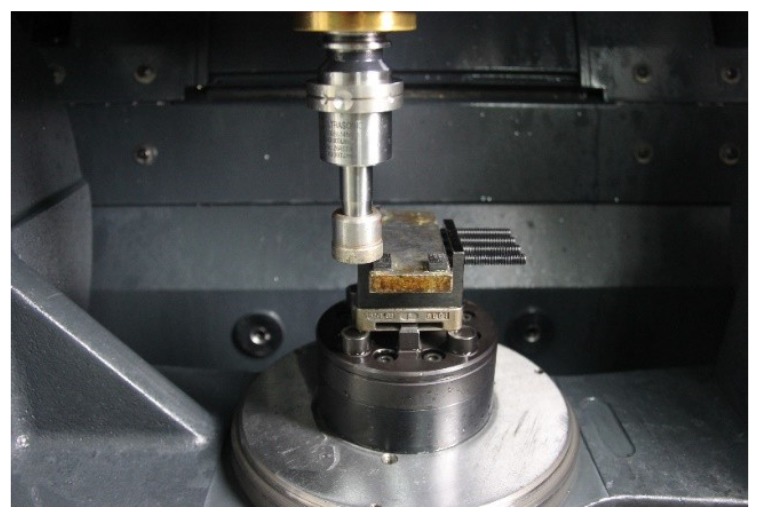
Test devices for ultrasound-assisted cutting with a single abrasive particle.

**Figure 3:**
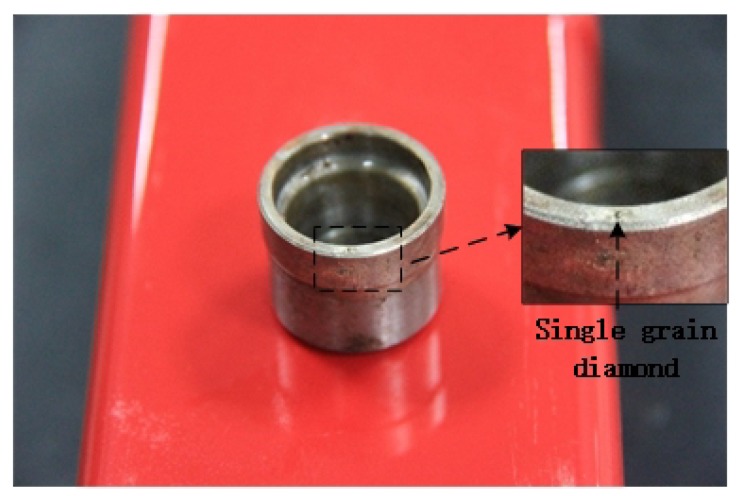
Ultrasound-assisted cutting tool with a single abrasive particle.

As shown in Figure 2, the test was performed on a vertical high-speed machining center (DMG Ultrasonic 20 Linear, DMG MORI, Bielefeld, Germany). As shown in Figure 3, a single diamond abrasive particle is brazed on the end face of a cup-shaped diamond grinding wheel base, which is made of low carbon steel (Type 45 steel), its diameter is ∅24 mm, the wall thickness is 2 mm, the diameter of the diamond abrasive 91 μm, as a cutting tool with a single abrasive particle. The particle size of the diamond abrasive particle is 40/50 mesh. During ultrasound-assisted cutting, the resonant frequency of the tool is 21.2 kHz and the end amplitude is approximately 4 μm. After selecting multiple sets of experimental parameters for the experiment, a set of experimental parameters was selected based on the experimental results and experience, under which the cutting force and temperature values were lower. The cutting parameters are as follows: the spindle speed *n* is 1000 rpm, the grinding depth $a_{p}$ is 10 μm, and the workpiece speed $V_{w}$ is 100 mm/min.

### 2.3. Observation Method

A scanning electron microscope (Hitachi S—3400N II, Hitachi, Tokyo, Japan) was used to observe the surface morphology of the sample cut by single abrasive particle, and the sample was sprayed with gold before observation.

## 3. Result and Discussion

### 3.1. Failure Mode of the Sic Matrix after Ultrasound-Assisted Grinding on C/SiC Composites

**Figure 4:**
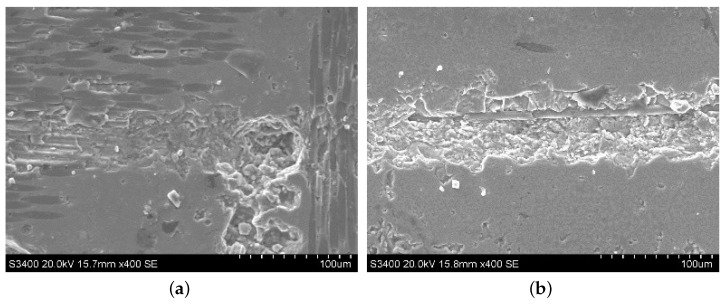
Typical surface morphologies of the SiC region in C/SiC composite after ordinary cutting and ultrasound-assisted grinding. (**a**) is ordinary grinding and (**b**) is ultrasound-assisted grinding.

Typical machined surface morphologies of the SiC region in C/SiC composite after ordinary cutting and ultrasound-assisted cutting are observed by SEM as shown in Figure 4. Due to ultrasound vibration, the diamond abrasive particle hammered the SiC surface periodically, and the hammering pits generated accordingly, resulting in the fact that the SiC surface has brittle spalling, which will also make the generation and propagation of cracks in the workpiece material easier, and the grinding force during the grinding process decreases. At the same time, compared with ordinary grinding, intensive hammering also makes the SiC region peel off more easily in ultrasound-assisted grinding. Therefore, it can be seen that the abrasive particle vibrates up and down under ultrasound vibration, which changes the motion form of the abrasive particle and weakens the cutting effect of the tool on the surface during processing.

### 3.2. Failure Modes of Carbon Fibers with Different Fiber Orientation Angles during Ultrasound-Assisted Grinding of C/Sic Composites

The matrix in C/SiC composites employs a hard and brittle material, and it is isotropic in the macro, so it is easier to analyze the material damage and removal process of the matrix. The carbon fiber layer is the part which mainly withstands external forces and made up of unidirectional non-woven cloth laid at different angles. It has obvious anisotropy and the fiber failure modes under different fiber orientation angle conditions are different. Anisotropy of the carbon fiber layer has an impact on the cutting force and surface morphology, so it is necessary to analyze failure modes of the carbon fiber with different fiber orientation angles.

**Figure 5:**
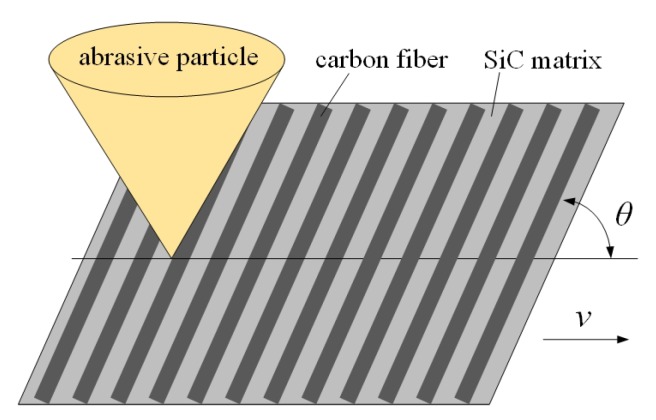
Diagram of a fiber orientation angle.

The definition of fiber orientation angle is shown in Figure 5, and the angle θ between the velocity orientation of the abrasive particle and the fiber orientation is defined as the fiber orientation angle.

#### 3.2.1. The Fiber Orientation Angle at $0^{\circ }$

When the tool cuts the fiber at a certain speed *v*, the main cutting force applied on the fiber is *F*. The main cutting force is caused by the interaction between the rake face of the tool and the material. Therefore, the direction of main cutting force is perpendicular to the rake face of the tool. However, due to the influence of fiber orientation angle, there are angles between the main cutting force and motion direction of the tool as the main cutting force and the fiber layer plane.

**Figure 6:**
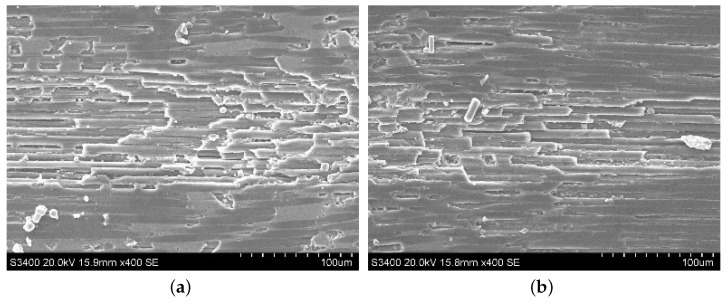
Typical morphologies after cutting C/SiC composites at $0^{\circ }$. (**a**) is ordinary cutting and (**b**) is ultrasound-assisted grinding.

**Figure 7:**
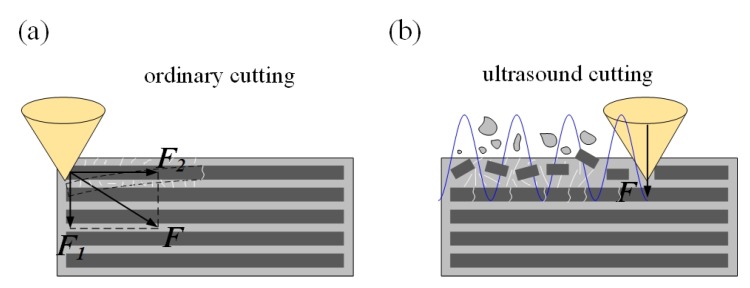
Comparison of material removal mechanisms between ordinary cutting and ultrasound cutting when the fiber orientation angle is $0^{\circ }$. (**a**) is ordinary cutting and (**b**) is ultrasound cutting.

**Figure 8:**
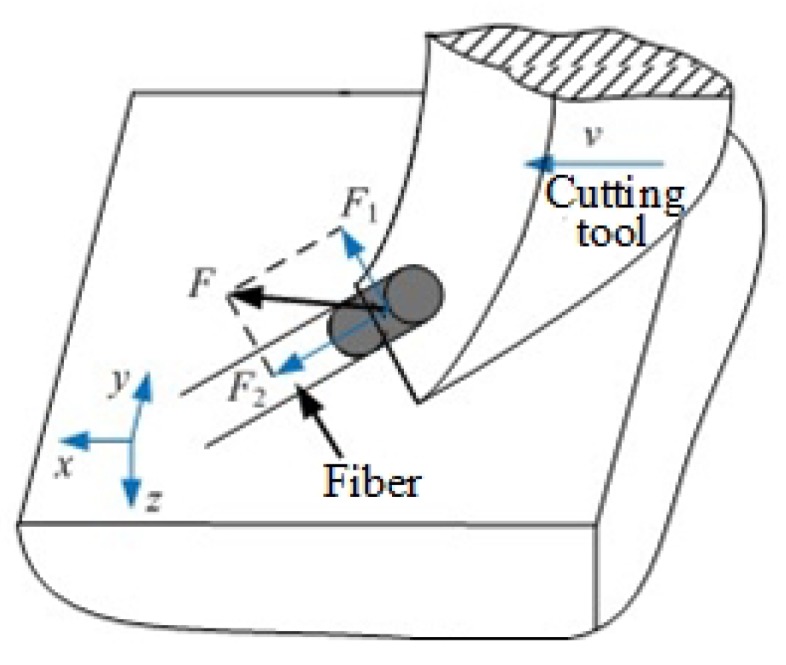
Analysis of a single fiber cutting force when fiber orientation angle is $0^{\circ }$.

The surface morphologies of ordinary cutting and ultrasound cutting when the fiber orientation angle is $0^{\circ }$ are shown in Figure 6. It can be seen that carbon fibers break along the cutting direction, and the fiber fractures are more intensive under the ultrasound condition. When the fiber orientation angle is $0^{\circ }$, the forces applied on the fibers are shown in Figure 7. In addition, Figure 8 shows the analysis of a single fiber cutting force when the fiber orientation angle is $0^{\circ }$. The main cutting force *F* generates a cutting force $F_{1}$ perpendicular to the fiber direction and a cutting force $F_{2}$ parallel to the fiber direction. $F_{1}$ is perpendicular to the direction of the fiber layer and points to the surface of the material. $F_{2}$ subjects the fiber to compressive stress. When the fiber orientation angle $\theta =0^{\circ }$, the single-particle cutter peels the carbon fiber from the silicon carbide matrix in the cutting area. The stripping process is smooth, the material in the cutting area is completely removed, and the cross-section is clear and tidy. This is because the tool moves in the same direction as the fiber. The tool can easily cut into the material and separate the carbon fiber from the silicon carbide matrix. Because the bonding strength between the carbon fiber and the silicon carbide matrix is much less than the shear strength of the carbon fiber, the carbon fiber is more easily peeled along the direction of the cutter movement. This form of cutting deformation can be called an interlayer peeling type. Under ordinary cutting condition, as shown in Figure 7, the main cutting force *F* can be divided into a cutting force $F_{2}$ parallel to the fiber orientation and a cutting force $F_{1}$ perpendicular to the fiber orientation. When applied the cutting force, the carbon fibers bend under pressure. As the abrasive particle moves forward, the fibers undergo strong deformation and eventually break in front of the abrasive particle at a certain distance. While under the condition of ultrasound cutting, as shown in Figure 7b, under the high-frequency strike of abrasive particle, the carbon fibers are cut into small pieces by the shear stress, and fracture is located under the abrasive particle.

#### 3.2.2. The Fiber Orientation Angle at $90^{\circ }$

**Figure 9:**
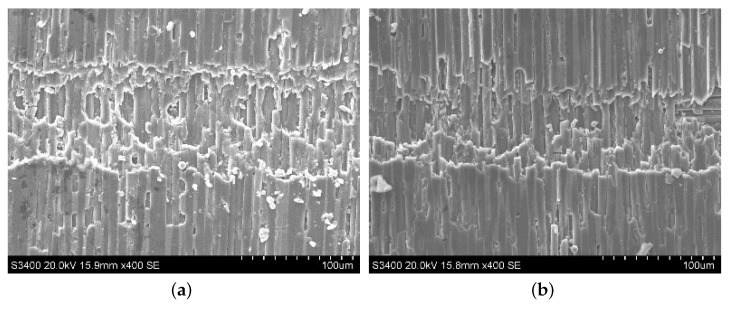
Typical morphologies after cutting C/SiC composites at $90^{\circ }$. (**a**) is ordinary cutting and (**b**) is ultrasound-assisted grinding.

**Figure 10:**
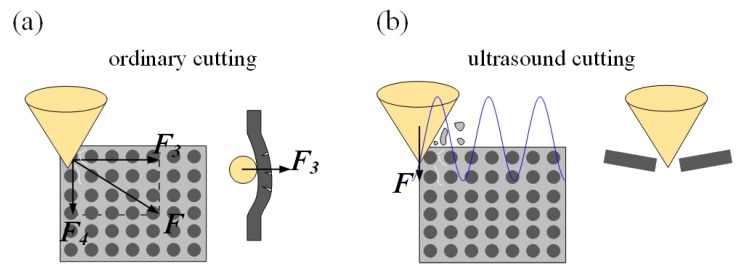
Comparison of material removal mechanisms between ordinary cutting and ultrasound cutting when the fiber orientation angle is $90^{\circ }$. (**a**) is ordinary cutting and (**b**) is ultrasound cutting.

**Figure 11:**
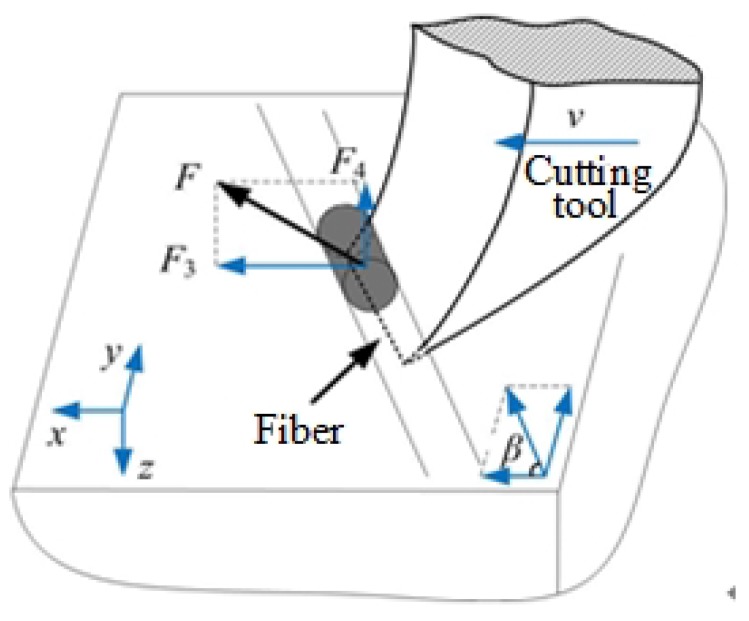
Force analysis of a single fiber during the cutting process when the fiber orientation angle is $90^{\circ }$.

Surface morphologies of ordinary cutting and ultrasound cutting when the fiber orientation angle is $90^{\circ }$ are shown in Figure 9. It can be seen that, under ordinary cutting conditions, the scratch width is larger than that of ultrasound cutting, which illustrates that, under ordinary cutting conditions, the fiber breaks are farther away from the abrasive particle than that of ultrasound cutting. When the fiber orientation angle is $90^{\circ }$, forces applied on the fibers are shown in Figure 10 and the force analysis of a single fiber during cutting is shown in Figure 11. Under ordinary cutting condition, the main cutting force *F* is perpendicular to the fiber axis, and can be divided into a cutting force $F_{3}$ on the plane of the fiber layer and a cutting force $F_{4}$ perpendicular to the plane of the fiber layer. The forces and deformations of the fibers on both sides of the tool are the same. The fibers may crack at the contact position and both sides of the tool. As the abrasive particle moves forward, the fibers bend and break, as shown in Figure 10a. Under the condition of ultrasound cutting, due to the high-frequency strike of the abrasive particle, the fibers are cut at the position where they contact the abrasive particle, as shown in Figure 10b.

#### 3.2.3. The Fiber Orientation Angle at $45^{\circ }$

**Figure 12:**
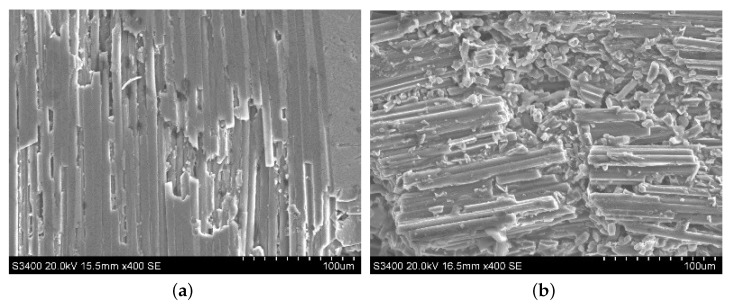
Typical morphologies after cutting C/SiC composites at $45^{\circ }$. (**a**) is ordinary cutting and (**b**) is ultrasound-assisted grinding.

**Figure 13:**
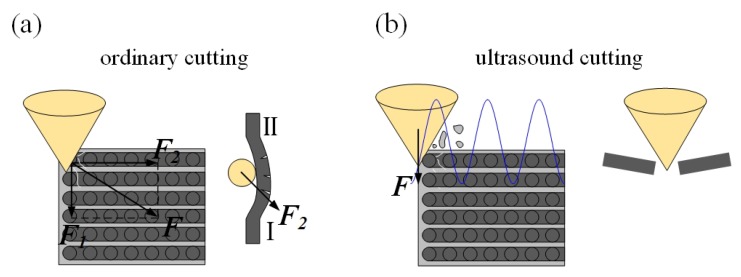
Comparison of material removal mechanisms between ordinary cutting and ultrasound cutting when the fiber orientation angle is $45^{\circ }$. (**a**) is ordinary cutting and (**b**) is ultrasound cutting.

**Figure 14:**
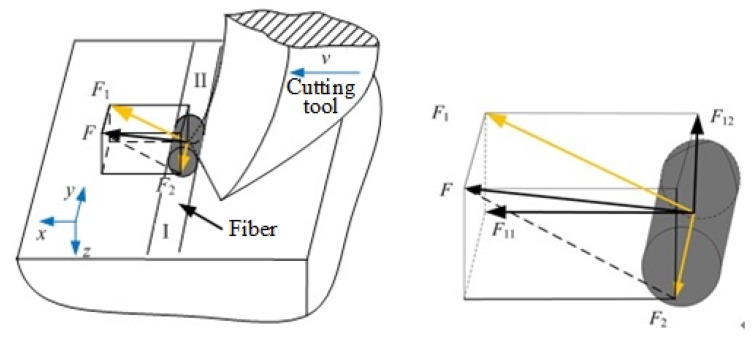
Force analysis of a single fiber during the cutting process when the fiber orientation angle is $45^{\circ }$.

When the fiber orientation angle is $45^{\circ }$, surface morphologies of ordinary cutting and ultrasound cutting are shown in Figure 12. It can be seen that, under ordinary cutting conditions, the carbon fibers at the scratch position peel off from the matrix, and the fracture region concentrates on both sides of the scratch, with a lot of uncut carbon fibers in the middle of the scratch. Under ultrasound conditions, the carbon fibers at the scratch position are cut into small pieces, forming a large amount of debris. When the fiber orientation angle is $45^{\circ }$, forces applied on the fibers are shown in Figure 13 and the force analysis of a single fiber during cutting is shown in Figure 14. The fibers can be divided into two parts, namely I and II. It can be seen that the cutting force $F_{2}$ parallel to the fiber direction makes the force applied to the I part of the fiber be compressive stress, while the force applied to the II part of the fiber is tensile stress. At the same time, the cutting force $F_{1}$ perpendicular to the fiber direction can be decomposed into a force $F_{11}$ in the plane of the fiber direction layer and a force $F_{12}$ perpendicular to the fiber direction layer. As the abrasive particle moves forward, the fibers in front of the abrasive particle undergo bending deformation. Since carbon fibers are brittle material, minor deformation can cause cracks. The cracks on the tensile stress side tend to expand, and it is more difficult for the cracks on the compressive stress side to expand. Therefore, the failure of carbon fibers with the fiber orientation angle of $45^{\circ }$ is mainly caused by cracks and expansion on the tensile stress side of the fibers during the bending deformation, which then leads to fracture.

It can be seen from Figure 6, Figure 9, and Figure 12 that, after ordinary cutting and ultrasound-assisted cutting, the fiber regions of the material both have stepwise breaks. Fibers under ordinary cutting are pulled out totally and break, while fibers under ultrasound-assisted cutting process are broken into pieces, and the affected area and damage degree are slightly lower than those of ordinary cutting. This fracture characteristic is similar to that of the SiC region, and both are related to the up-and-down vibration of the abrasive particle under ultrasound vibration, which is the main reason that the cutting force of ultrasound-assisted grinding is smaller than that of ordinary grinding. Under ordinary cutting condition, cutting quality with the fiber orientation angle of $0^{\circ }$ is better than that of $90^{\circ }$, and even much better than that of $45^{\circ }$, while, for ultrasound cutting, under different fiber orientation angle conditions, all the scratches show that the carbon fibers are fully broken, and the scratch quality tends to be consistent.

**Figure 15:**
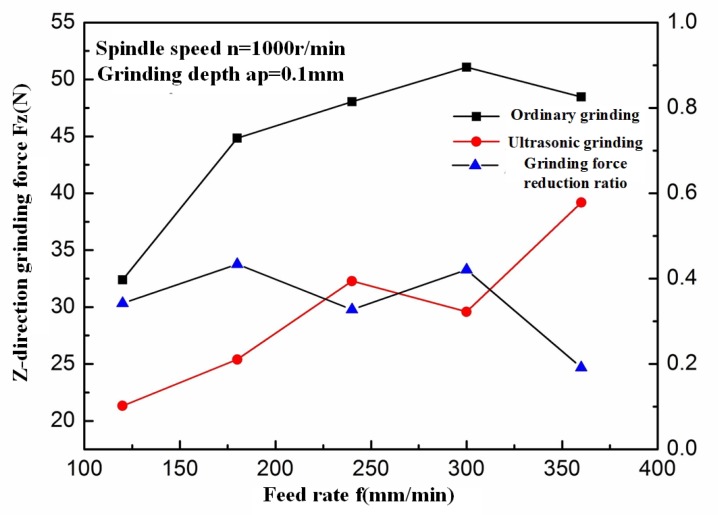
Comparison of ultrasonic grinding and ordinary grinding force at different feed speeds.

At the same time, we compared the grinding force under different feed speeds of ordinary grinding and ultrasonic vibration-assisted grinding. It can be seen from Figure 15 that, under the condition of ultrasonic vibration-assisted grinding, the component forces in all three directions are smaller than the grinding forces in the ordinary grinding condition. The grinding forces are reduced to varying degrees, and the maximum reduction ratio is about 60%.

### 3.3. Morphology Analysis of Abrasive Debris during Ultrasound-Assisted Grinding of C/SiC Composites

#### 3.3.1. Single Abrasive Particle Cutting Model of C/SiC Composites

**Figure 16:**
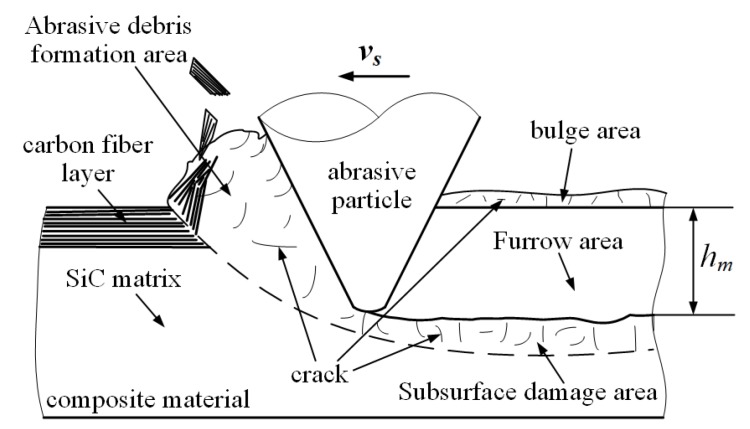
Two-dimensional cutting model of single abrasive particle for C/SiC composites.

In order to reveal the removal mechanism of C/SiC composites, a two-dimensional cutting model was established based on the cutting effect of single abrasive particle on C/SiC composites, as shown in Figure 16.

Combining the material preparation process with the actual machining process, it is assumed in the model that the carbon fiber layer is always on the surface of the cutting area. It can be known from the simplified process of the above model that the cutting depth of the abrasive particle in Figure 16 is the maximum undeformed abrasive debris thickness $h_{m}$ of vertical-axis plane grinding, and the cutting speed of the abrasive particles is $v_{s}$. The cutting area can be divided into four deformation areas, namely abrasive debris formation area, subsurface damage area, furrow area, and bulge area.

Material deformation process in the abrasive debris formation area is as follows: when the abrasive particle begins to cut the C/SiC composites, the cutting edge contacts with the carbon fiber layer and the SiC matrix layer at the same time. The SiC matrix is a brittle material, so cracks, deformations, and fractures occur firstly. The short fibers play a role as supporting. Therefore, in the initial stage of the cutting process, the abrasive debris generated by the SiC matrix layer still maintains integrity and has the trend of outflow in front of the blade. As the abrasive particle moves on, the deformation of the carbon fiber layer increases, and, at the same time, the stress increases under the squeezing of the SiC matrix material underneath. Shear fracture occurs when the strength limit is reached, and the carbon fiber layer generates broken pieces. Due to suddenly released stress, the SiC matrix layer in front of the cutting edge also breaks up into abrasive debris with short fibers.

The subsurface damage area is below the surface already cut by the abrasive particle. A large number of microcracks are distributed in this region. These microcracks are formed by the imprinting of the abrasive particle on the hard and brittle SiC matrix material. Imprinted cracks of brittle materials are generally divided into median cracks (roughly perpendicular to the cutting surface) and transverse cracks (roughly parallel to the cutting surface). These cracks are the main forms of subsurface damage during grinding on C/SiC composite. The subsurface damage has a bad effect on anti-fatigue and anti-wear properties of the surface, but it can only be reduced as much as possible, and cannot be completely eliminated.

The furrow area is a processed surface formed behind the abrasive particle after the abrasive particle interacts with the workpiece material to form abrasive debris. Because there are a lot of abrasive particles randomly distributed on the grinding wheel, under the cutting of all the abrasive particles, the furrow areas formed by each abrasive particle are connected and overlapped with each other, and the material is removed.

The bulge area exists on the side of the furrow area, and is higher than the original surface of the workpiece. The formation of the bulge area is because when the abrasive particle cuts the material, the material becomes abrasive debris and flows out not only from the front of the cutting edge, but also from the side of the cutting edge. This part of material lacks sufficient deformation and no abrasive debris peels off. It remains on the surface, which has a lot of microcracks, and the strength is poor. Generally, bulge material always remains at the boundary of the milling groove. Bulge of the SiC matrix is likely to cause edge breakage, and bulge of the carbon fiber forms burrs. Both of the two defect modes are bad for improving the processing quality.

#### 3.3.2. Morphology Comparison of the Abrasive Debris at Different Cutting Depths

**Figure 17:**
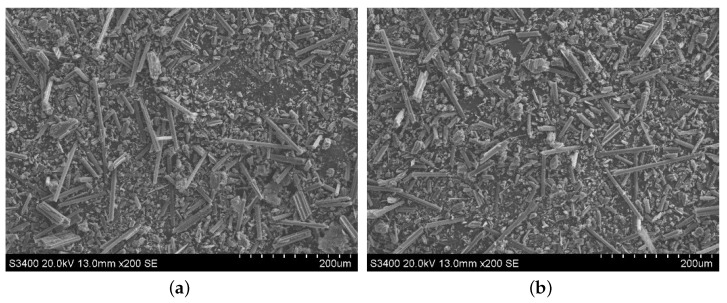
Morphologies comparison when $a_{p}$ is 0.1 mm. (**a**) is ordinary cutting and (**b**) is ultrasound- assisted cutting.

**Figure 18:**
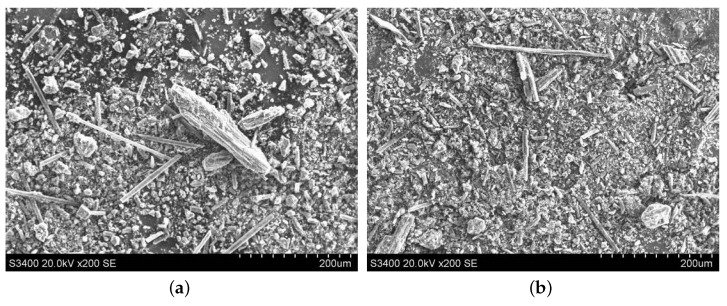
Morphologies comparison when $a_{p}$ is 0.2 mm. (**a**) is ordinary cutting and (**b**) is ultrasound- assisted cutting.

**Figure 19:**
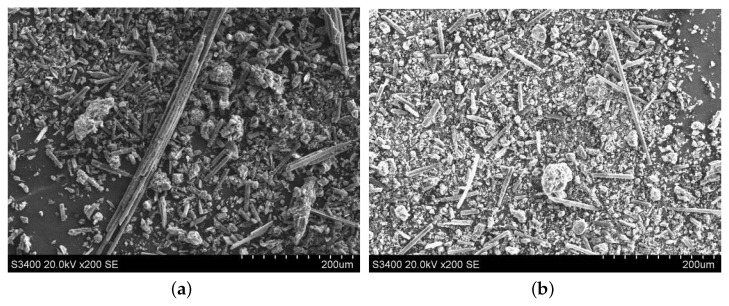
Morphologies comparison when $a_{p}$ is 0.3 mm. (**a**) is ordinary cutting and (**b**) is ultrasound- assisted cutting.

**Figure 20:**
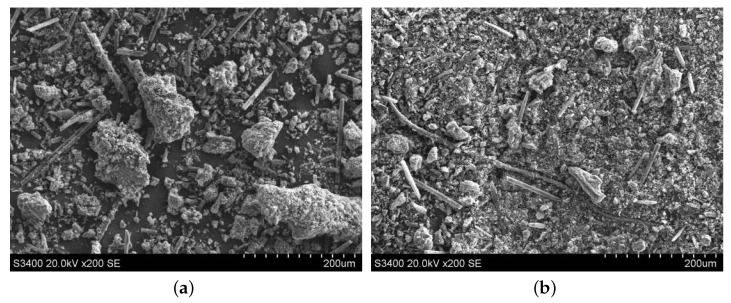
Morphologies comparison when $a_{p}$ is 0.5 mm. (**a**) is ordinary cutting and (**b**) is ultrasound- assisted cutting.

The abrasive debris of ordinary cutting and ultrasound cutting under different cutting depth conditions was collected, and the scanning electron microscopy was used to observe the morphologies of the abrasive debris under different conditions, as shown in Figure 17, Figure 18, Figure 19 and Figure 20. It can be seen that the abrasive debris generated by grinding on C/SiC composites can be roughly divided into two types: abrasive debris formed by the SiC matrix and abrasive debris formed by the carbon fibers. Under ordinary cutting conditions, the abrasive debris formed by the SiC matrix changes from powder to piece with the increase of cutting depth. While under the condition of ultrasound cutting, the abrasive debris formed by the SiC matrix basically remains powdery, which was not affected much by the change of $a_{p}$. Under ordinary cutting condition, as the cutting depth of the abrasive particle increases, the abrasive debris formed by the carbon fibers changes from short strips to long strips. While under ultrasound cutting conditions, morphology of the abrasive debris formed by the carbon fibers roughly remains short bar.

From the failure mode analysis of the C/SiC composite material during the cutting process in the previous section, it can be known that, under ordinary cutting conditions, removal of the material is achieved by matrix extrusion fracture and fibers bending fracture. During the material removal process, the abrasive particle is in constant contact and has friction with the matrix and fibers, and the fracture energy of the material is completely provided by the abrasive particle. While in the ultrasound cutting process, removal of the material is achieved by the strike and shear under high frequency vibration of the abrasive particle on the matrix and fibers. The vibration energy of the abrasive particle is additionally provided by the ultrasound power source. Due to the intermittent contact of the abrasive particle and the material, and the bending deformation of the fibers before breaking is small, the friction during the cutting process is significantly smaller than that of ordinary cutting. Therefore, the cutting force of ultrasound vibration is significantly smaller than that of ordinary cutting, and wear of the abrasive particle is also smaller than that of ordinary cutting. In addition, under the condition that the cutting depth increases, the abrasive particle morphology of ultrasound cutting changes little, indicating that ultrasound vibration processing can operate smoothly within a wide range of processing parameters, and the efficiency is significantly improved compared with ordinary grinding.

## 4. Conclusions

In this paper, a cutting method with single abrasive particle is adopted to study the material removal mechanism of C/SiC composites during the ultrasound vibration-assisted grinding process. A two-dimensional cutting model of a single abrasive particle is established to analyze the typical characteristics and abrasive debris forming process under different conditions. By comparing with the experimental result and SEM images, the material removal mechanism of C/SiC composites was revealed. The main conclusions are as follows:

1. With different fiber orientation angles, the main failure mode of the carbon fibers by ordinary cutting is bending fracture, while the main failure mode of the carbon fibers by ultrasound cutting is shear fracture;
2. With the increase of cutting depth, the sizes of the SiC matrix piece and carbon fibers bar by ordinary cutting tend to increase to be larger and longer. However, due to periodic high-frequency strike of the abrasive particle on the matrix, the SiC matrix is more fully broken under ultrasound cutting conditions remaining powdery and the abrasive debris of the carbon fibers is short bar, which are not much affected by the change of $a_{p}$;
3. Ultrasound vibration grinding of C/SiC composites has the advantages of reducing grinding forces; under the conditions of ultrasonic vibration-assisted grinding, the grinding force is reduced to varying degrees, and the maximum reduction ratio reaches about 60%, which means that ultrasonic vibration is more effective for the material removal.
